# Prognostic biomarker discovery based on proteome landscape of Chinese lung adenocarcinoma

**DOI:** 10.1186/s12014-023-09449-2

**Published:** 2024-01-05

**Authors:** Yuqi Huang, Sheng Ma, Jun-Yu Xu, Kun Qian, Yaru Wang, Yi Zhang, Minjia Tan, Ting Xiao

**Affiliations:** 1 State Key Laboratory of Drug Research, Shanghai Institute of Materia Medica, Chinese Academy of Sciences, Shanghai, 201203 China; 2 University of Chinese Academy of Sciences, Beijing, China; 3 State Key Laboratory of Molecular Oncology, Department of Etiology and Carcinogenesis, National Cancer Center/National Clinical Research Center for Cancer/Cancer Hospital, Chinese Academy of Medical Sciences and Peking Union Medical College, Beijing, 100021 China; 4 Department of Thoracic Surgery, Xuanwu Hospital, Capital Medical University, Beijing, 100053 China; 5 Zhongshan Institute for Drug Discovery, Shanghai Institute of Materia Medica, Chinese Academy of Sciences, Guangdong, 528400 China

**Keywords:** Lung adenocarcinoma, Proteome, Benign lung disease cases, Solid pathological subtype, Prognostic biomarker

## Abstract

Despite recent innovations in imaging and genomic screening promotes advance in diagnosis and treatment of lung adenocarcinoma (LUAD), there remains high mortality of LUAD and insufficient understanding of LUAD biology. Our previous study performed an integrative multi-omic analysis of LUAD, filling the gap between genomic alterations and their biological proteome effects. However, more detailed molecular characterization and biomarker resources at proteome level still need to be uncovered. In this study, a quantitative proteomic experiment of patient-derived benign lung disease samples was carried out. After that, we integrated the proteomic data with previous dataset of 103 paired LUAD samples. We depicted the proteomic differences between non-cancerous and tumor samples and among diverse pathological subtypes. We also found that up-regulated mitophagy was a significant characteristic of early-stage LUAD. Additionally, our integrative analysis filtered out 75 potential prognostic biomarkers and validated two of them in an independent LUAD serum cohort. This study provided insights for improved understanding proteome abnormalities of LUAD and the novel prognostic biomarker discovery offered an opportunity for LUAD precise management.

**Supplementary Information:**

The online version contains supplementary material available at 10.1186/s12014-023-09449-2.

## Introduction

In recent years, despite the incidence and mortality rate of lung cancer decreased gradually in the whole world [1], lung cancer remains the most common cancer in China and the leading cause of cancer-related death worldwide [1–3]. Non-small cell lung cancer (NSCLC) is a major subtype of lung cancer (about 80–85%). In further subdivision, lung adenocarcinoma is most dominant histological NSCLC phenotypes, accounting for approximately 40% of histologic type of whole lung cancer types [4].

According to the IASLC/ATS/ERS classification system, invasive adenocarcinomas are usually classified to lepidic, acinar, papillary, micropapillary, solid subtypes by predominant histologic pattern [5] (defined as LEP, ACI, PAP, MIP and SOL separately in the following paper). Among them, SOL tumors were associated with poor OS for patients receiving adjuvant chemotherapy and poor DFS for stage III/IV patients without radiotherapy [6]. Genomics alterations of predominant histologic subtypes were described in previous study, in which SOL and MIP tumors harbor higher tumor mutational burden. Higher BRAF-V600E mutation frequency was observed in SOL/MIP tumors. In addition, significant genomic alterations of Myc, p53 and Wnt pathway were identified in SOL/MIP tumors [7]. However, it remains unclear about the proteomic characteristics of the malignant solid pathological LUAD subtype.

Within invasive techniques for lung cancer diagnosis, transthoracic fine needle aspiration and biopsy are widely utilized [8]. Meantime, some non-invasive imaging tools such as CT and PET have already played important roles in diagnosis and staging of NSCLC [8–10]. However, limited by cost and potential radiation injury, frequent CT/PET examination in follow-up disease monitoring and risk assessment may be improper. As supplement to current risk assessment system, exploration of non-invasive new biomarkers with high sensitivity and high specificity appear particularly important. In recent years, applications of genomic biomarker made great progress in NSCLC subtyping and therapeutic decision making [11], which have contributed to the reduction of population-level mortality of NSCLC in US from 2013 to 2016 [12]. Although the genomic characteristics of LUAD show disparities between different ethnic groups [13–15], testing for dominant genomic level variation event such as EGFR mutation, ALK rearrangement, ROS arrangement and BRAF V600 mutation status was recommended in clinical practice by both NCCN Guidelines and Pan-Asian adapted Clinical Practice Guidelines [8, 16]. Nevertheless, due to the limited application range, the proportion of patients who benefited from genome-driven treatment was low (4.90% in US, 2018) [17]. As the results of all kinds of downstream processes (alternative splicing, RNA processing, translation, miRNA, etc.) of the genome, the proteome profiles, with high complexity and dynamics, connect the genotype to disease phenotype [18]. Therefore, exploration of biomarkers at proteome level could help improve the understanding of tumor biology and promote their clinical application.

In this study, we performed a label-free quantitative proteomic analysis of 14 benign lung disease cases, including pneumothorax, bronchiectasis etc. Next, we integrated the proteomic data from these non-cancerous lung disease samples with our previously acquired 103 paired LUAD with nearest adjacent tissue (NAT) samples, underlying the molecular characteristics of early-stage LUAD and diverse LUAD pathological subtypes, especially for the malignant solid pathological LUAD subtype. Furthermore, we used this large-scale LUAD proteomic cohort with their survival information to explore novel protein prognostic biomarkers and verified two of the prognostic biomarkers in an independent serum cohort. Our study provided important proteomic information of pathological LUAD subtypes, indicating the potential clinical utilization value of proteomic outcome.

## Results

### Proteome landscape

**Fig. 1:**
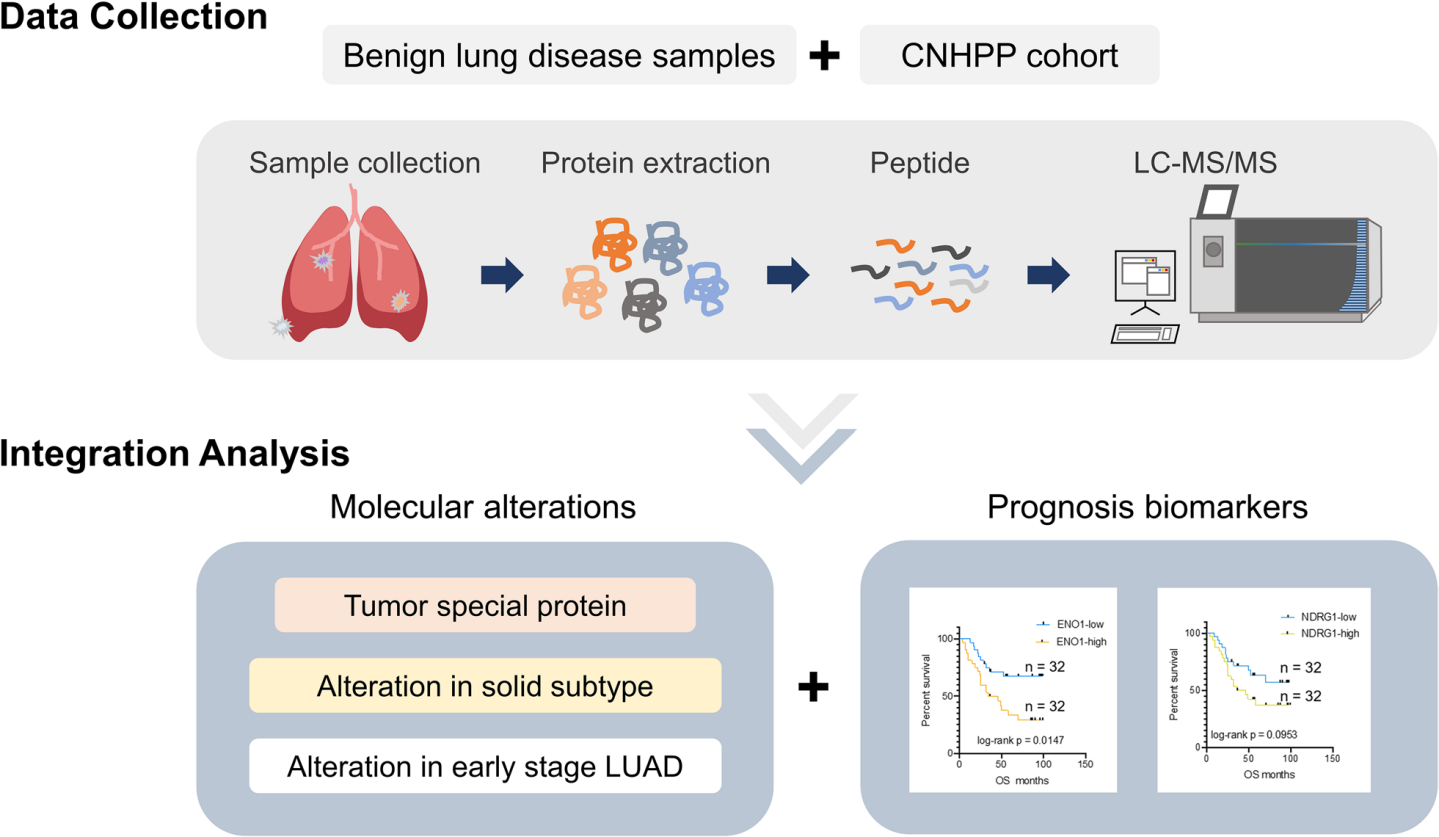
Label-free based proteome landscape of benign lung disease, LUAD and NATs. Workflow of MS/MS data collection and analysis

We conducted the label-free quantitative proteomic experiment and analysis of 14 benign lung disease samples derived from pneumothorax (n = 3), bronchiectasis (n = 2), benign lung hyperplasia (n = 6) and granulomatous inflammation (n = 3) patients (Additional file 1: Fig S1A) according to the method used in our previous LUAD proteome analysis [19]. After that, we combined the proteomic data of 14 benign lung disease cases with our previous 103 paired LUAD proteome (Chinese Human Proteome Project, CNHPP dataset), and then re-normalized them for further integrative analysis (Fig. 1, Additional file 6: Table S1). We evaluated the correlation between the proteomic data in CNHPP dataset and that re-normalized in our current matrix to avoid bias introduced during data normalization process. For each LUAD and NAT sample, the proteome profiles normalized in CNHPP study and in this study shown high consistency (*p* < 0.05, r > 0.9999) (Additional file 1: Fig S1B). In addition, the protein abundances of all samples met the criteria of a same unimodal distribution after quantile normalization (Additional file 1: Fig S1C). As a result, we identified a total of 11,726 proteins (11,234  unique gene names) for all 220 samples. For tumor samples, the average number of quantified proteins (summed to gene levels) was 7,362. For NAT and benign lung disease samples, the average number of quantified proteins was 5,946 and 5,721, respectively (Additional file 1: Fig S1D). After that, we assessed the protein subcellular localization of the identified proteins, and the results showed that these proteins were located primarily at nuclear, cytosol, serum membrane and mitochondria. Meantime, a small part of the identified proteins was in the components of centrosome, peroxisomes, and lysosomes. In addition, the identified protein numbers of each subcellular component were higher in the tumor samples in comparison with that observed in NAT and benign lung disease samples (Additional file 1: Fig S1E).

### Proteomic features of tumor and non-tumor sample

**Fig. 2:**
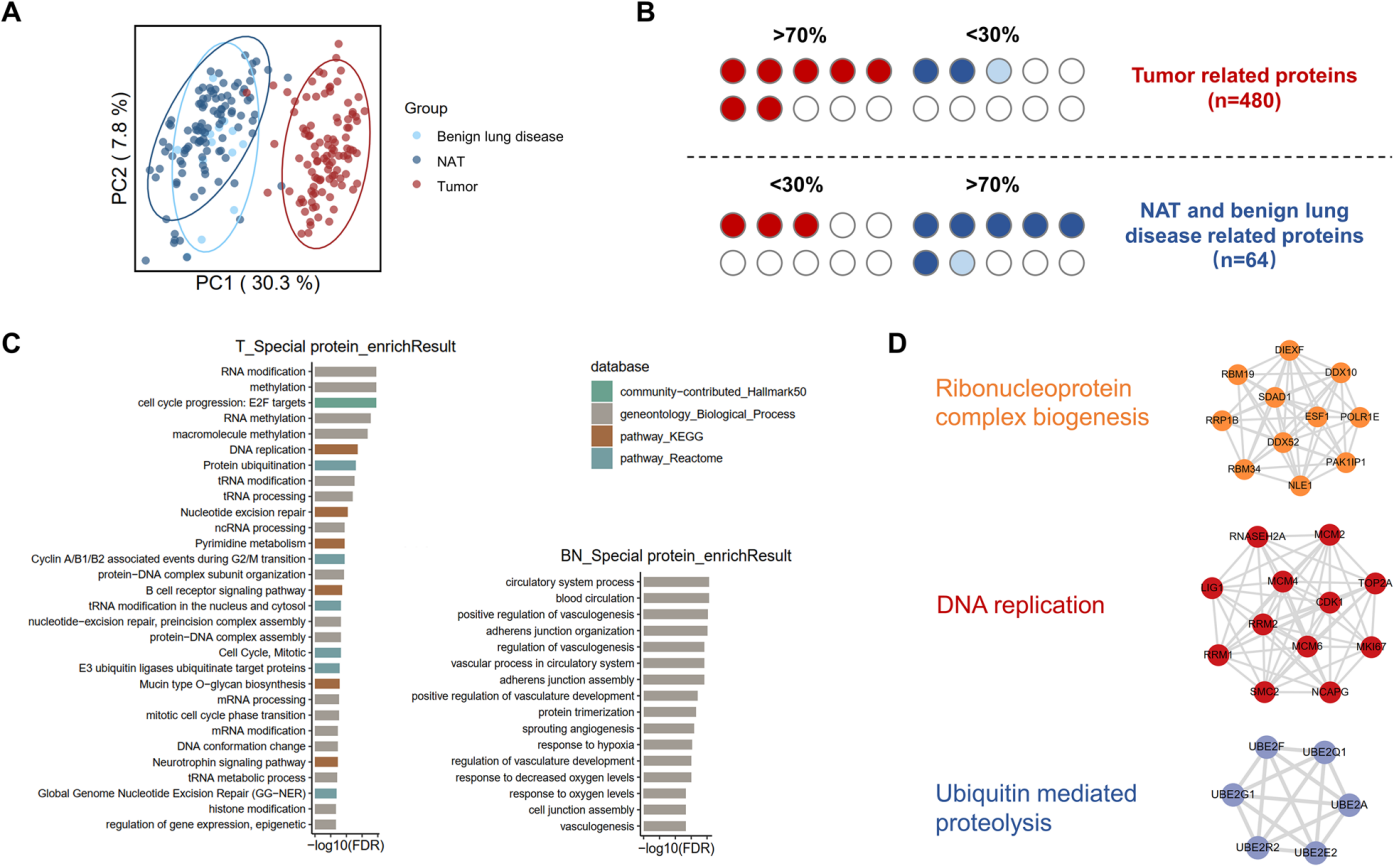
Proteomic features of LUAD and non-tumor samples. **A** Principal component analysis for benign lung disease, NATs and LUAD samples. **B** Definition of LUAD related proteins, NAT and benign lung disease related proteins. **C** ORA pathway enrichment results for LRPs and NDRPs. **D** STRING protein–protein interaction network (top panel) and MCODE protein cluster analysis for LRPs (bottom panel)

We performed principal component analysis for the whole 220 samples. The tumor samples could be separated clearly with NATs and benign lung disease samples in principal component 1 which explained up to 30.3% variance of the normalized data matrix (Fig. 2A). Though there was little difference between NAT and benign lung disease samples in principal component analysis, we found some altered signaling pathways between these two groups through differential analysis. We noticed that proteins in sphingolipid signaling pathway, relaxin signaling pathway and apelin signaling pathway were up-regulated in benign lung disease when compared with both NAT and tumor groups. Meanwhile, ECM-receptor interaction pathway was up-regulated only in NAT group, which suggested a reduced function of ECM-receptor even in non-tumor lung disease (Additional file 2: Fig S2A). As described above, the number of proteins identified in tumor samples was much higher compared to that observed in non-tumor samples (NAT and benign lung disease samples), and the results showed that a part of proteins could be only identified in tumor samples. We firstly calculated the frequency of protein identification before missing value imputation, which provided abundant information about the biological differences of tumor and non-tumor samples. Therefore, we defined 480 proteins that identified in more than 70% tumor samples and less than 30% non-tumor samples as LUAD related proteins (LRPs) while 64 proteins that identified in more than 70% non-tumor samples and less than 30% non-tumor samples were defined as NATs and benign lung disease related proteins (NDRPs) (Fig. 2B). WebGestalt over-representation analysis demonstrated that oncogenic pathways related to RNA and histone epigenetic modification, cell cycle, nucleotide excision repair and protein ubiquitination *et.al* were significantly in LRPs, while pathways related to circulatory system and cell junction were enriched in NDRPs. This result indicated the activated signaling of cell proliferation and metastasis as well as the loss of cell adherent molecular in tumor tissues (Fig. 2C). Additionally, we constructed a functional association network of LRPs with STRING and extracted 3 major sub protein clusters through MCODE algorithm. Among them, Cluster 1 consisted of proteins belonging to ribonucleoprotein complex biogenesis pathway such as DEAD box (DDX) family proteins and RNA binding motif (RBM) family proteins. Cluster 2 consisted of proteins involved in DNA replication process such as mini chromosome maintenance (MCM) family proteins and ribonucleotide reductase regulatory subunit M1/2(RRM1/2) (Fig. 2D).

### Characteristics of solid pathological subtype

**Fig. 3:**
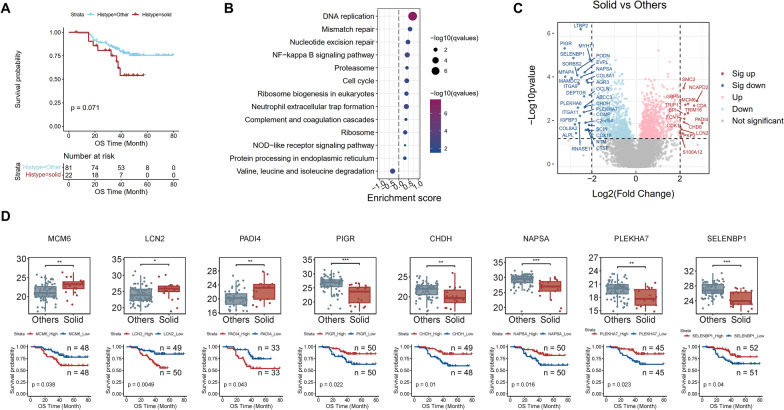
Characteristics of solid-predominant adenocarcinomas. **A** Kaplan–Meier curve of overall survival in SOL and non-SOL adenocarcinoma samples. **B** Pathway enriched in SOL or the rest tumor samples based on GSEA. Enrichment ratio > 0 indicated the pathway was enriched in SOL samples; Enrichment ratio < 0 indicated the pathway was enriched in the rest tumor samples. **C** Differentially expressed proteins between SOL and non-SOL adenocarcinoma samples. A criteria of p < 0.05 and abs (log2 fold change) > 2 was applied to define significantly up- or down- regulated proteins. **D** Differentially expressed proteins which were significantly correlated with prognosis. Top panel: protein expression level in SOL and non-SOL adenocarcinoma samples. Bottom panel: Kaplan–Meier curve of overall survival in samples with high expression (red line) and low expression (blue line) of the same protein presented in the top panel

Our dataset covered most clinical LUAD histologic subtypes including acinar-predominant (ACI, n = 49), papillary-predominant (PAP, n = 18), solid-predominant (SOL, n = 22), lepidic-predominant (LEP, n = 5) adenocarcinomas and mix subtype (n = 8) adenocarcinomas. ACI, PAP and LEP adenocarcinomas together with one sample without pathological information were defined as non-solid-predominant (non-SOL, n = 81). In our dataset, a tendency of poor prognosis was observed in SOL adenocarcinoma patients (p = 0.07, Fig. 3A), and this result was consistent with pervious study [6, 20].

To further understand biological characteristics of SOL, we evaluated the molecular difference between SOL and non-SOL adenocarcinoma samples in proteomic level. GSEA analysis showed that many cell proliferation related pathways were significantly up regulated in SOL adenocarcinomas, including DNA replication, mismatch repair, cell cycle, ribosome biogenesis, etc. Beyond that, NF-kappa B signaling pathway, a classic carcinogenic which could promote lung carcinoma progression [21, 22] was unveiled to be enhanced in SOL adenocarcinomas. Additionally, complement and coagulation cascade, neutrophil extracellular trap formation and NOD-like receptor signaling pathways were also up-regulated in SOL adenocarcinomas while valine, leucine and isoleucine degradation pathway was down-regulated (Fig. 3B). We identified 15 up-regulated proteins and 30 down-regulated proteins in SOL subtypes. In addition, for each protein, we calculated the median CERES score [23] across all LUAD cell lines to evaluate the gene dependency (Fig. 3C). Among these differentially expressed proteins, MCM6, LCN2 and PADI4 were highly expressed in SOL adenocarcinomas and the high expression of which was correlated with poor OS. MCM6, a DNA replication licensing factor, had median CERES score less than -0.6. Lipocalin-2 (LCN2) was previously unveiled to be elevated in human LUAD but able to counteracts LUAD development by maintaining antitumor immunity in the early pathogenesis of LUAD [24], indicating a complex role of LCN2 in the LUAD progress. Peptidyl arginine deiminase 4 (PADI4) was recently found to be involved in HIF-dependent transcriptional response to tumor vascularization and hypoxia through histone citrullination [25], which might explain its correlation to poor prognosis. Parallel to that, 5 proteins were expressed lower in SOL adenocarcinomas and the low expression of which was correlated with poor OS, including PIGR, CHDH, NAPSA, PLEKHA7 and SELENBP1(Fig. 3D).

### Common and special molecular alterations in entire and early stage LUAD samples

**Fig. 4:**
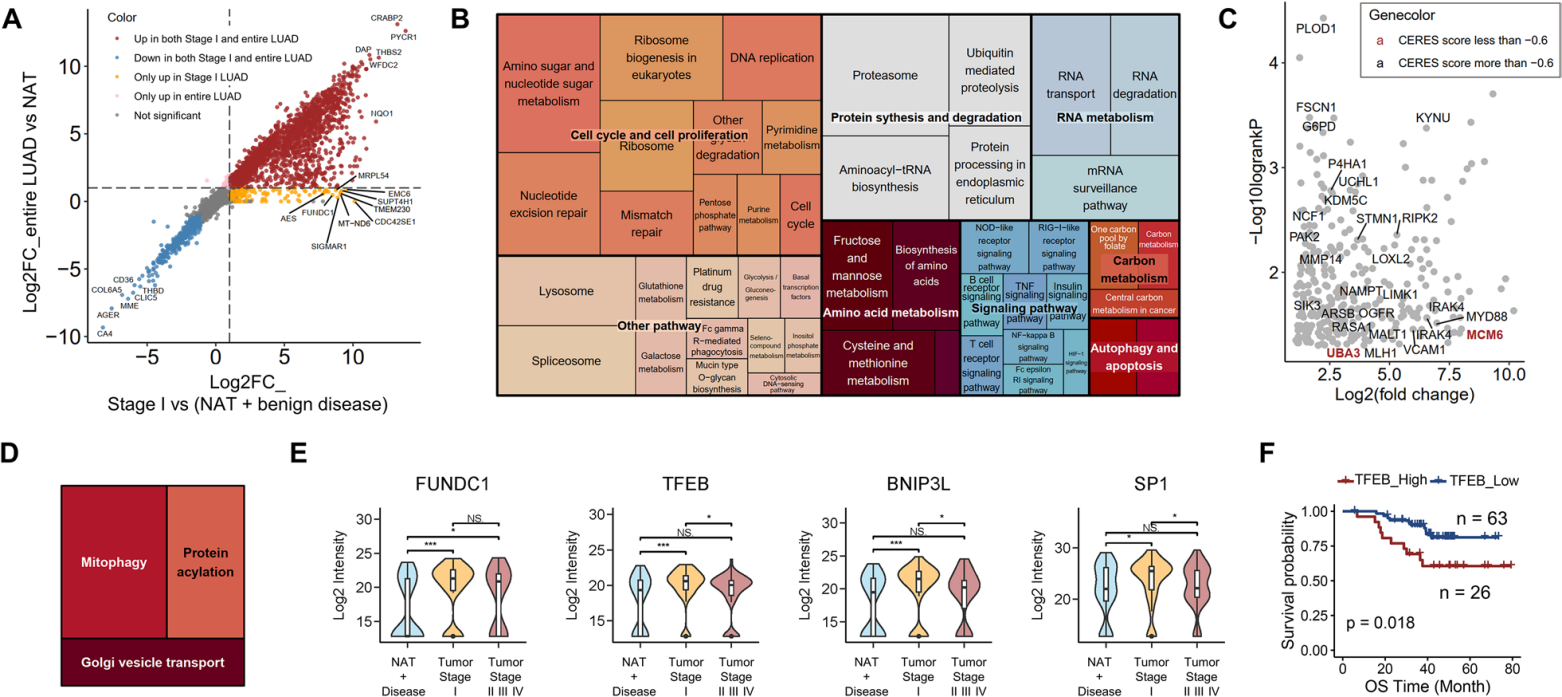
Shared and special molecular alterations in entire and early stage LUAD samples. **A** Comparison of fold-changes in early stage or entire LUAD samples to non-tumor samples. Red dots: proteins up-regulated (fold of change > 2, p < 0.05) in both early stage or entire LUAD groups. Blue dots: proteins down-regulated (fold of change < 1/2, p < 0.05) in both early stage or entire LUAD groups. Orange dots: proteins up-regulated (fold of change > 2, p < 0.05) in only early stage group. Pink dots: proteins up-regulated (fold of change > 2, p < 0.05) in only entire LUAD group. Grey dots: proteins without significant change (p > 0.05) in both early stage or entire LUAD groups. **B** KEGG pathways enriched in shared up-regulated proteins. **C** The log-rank p values and fold of changes of the shared up-regulated proteins. Proteins belonging to clinical successful or potential drug targets were labeled with gene names. **D** KEGG pathways enriched in Stage I unique up-regulated proteins. **E** Distributions of the expression level of four ‘Stage I unique up-regulated proteins’ belonging to mitophagy pathway in non-tumor, early stage and middle and late stage tumor groups. (*p < 0.05. **p < 0.01. ***p < 0.001.). **F** Kaplan–Meier curve of overall survival in samples with high expression (red line) and low expression (blue line) of TFEB

Although tumor node metastasis (TNM) Stage I LUAD patients who receive surgical resection have a high recovery rate, up to 20–30% of early-stage patients have a poor prognosis [26]. In the CNHPP study, we mainly analyzed the different molecular characteristics between early stage (stage I) LUAD patients with good or poor prognosis [19]. Considering the complexity and dynamics of protein expression during the progress of LUAD, we compared the differences between early stage or entire LUAD samples to non-tumor samples. A criteria of fold change > 2 and *p* < 0.05 was applied in filtering of significant up-regulated proteins (Fig. 4A). Most significant up-regulated proteins in comparison to non-tumor samples were shared (n = 3,414) in early stage or entire LUAD samples, and we defined them as “shared up-regulated proteins”. These proteins were enriched in pathways related with cell cycle, protein synthesis and degradation, RNA metabolism and apoptosis, etc. (Fig. 4A, B). We then visualized their survival information and added annotations for clinical utilized drugs or potential drug targets. Among these shared up-regulated proteins, 2-oxoglutarate 5-dioxygenase 1 (PLOD1) showed the minimum log-rank *p* value in our dataset (Fig. 4C). PLOD1 was also reported to be a prognostic biomarker for osteosarcoma [27]. According to Therapeutic Target Database (TTD) [28], PLOD1 is a potential drug target under clinical trial stage. For each protein related with poor prognosis, we calculated their median CERES scores. The results showed that MCM6 and NEDD9-activating enzyme E1C (UBA3), annotated as literature-reported targets, had median CERES scores less than -0.6, which prompted potential new therapeutic strategies (Fig. 4C). A small part of proteins was only up-regulated in early stage LUAD (n = 228, defined as ‘Stage I unique up-regulated proteins’ in the following paper) and the KEGG mitophagy pathway was enriched in these proteins (Fig. 4D). In mitophagy pathway, a total of seven proteins belonged to Stage I unique up-regulated proteins, among which TFEB, BNIP3L and SP1 were either statistical significantly up-regulated in Stage I when compared to middle and late stage tumor groups (Fig. 4E). In the contrast, no significant differences of protein expressions were observed between Stage I and Stage II/III/IV groups for FUNDC1, CALCOCO2, SRC and BCL2L1 (Additional file 3: Fig S3A).

Further analysis indicated that the high expression of FUNDC1 and TFEB was correlated to poor OS in our dataset (Additional file 3: Fig S3B; Fig. 4F). FUNDC1 is a mitochondrial outer membrane protein involved in hypoxia-induced mitophagy as a mitophagy receptor [29]. Transcription factor EB (TFEB), belonging to the microphthalmia family of transcription factors, is a master regulator of lysosomal biogenesis and autophagy [30, 31]. TFEB was previously reported to be associated with poor prognosis in NSCLC samples [32] and mediated lysosomal and autophagosomal biogenesis triggered by inhibition of p53 in lung cancer cells [33]. The findings indicated that mitophagy could be enhanced especially in early LUAD progress, and the abnormal activation of mitophagy may contribute to poor survival in LUAD. Besides, protein acylation and Golgi vesicle transport pathway were also enriched in Stage I unique up-regulated proteins (Fig. 4D).

In addition, we compared the early-stage upregulated proteins (early stage LUAD vs non-tumor) and late-stage upregulated proteins (late stage LUAD vs early stage LUAD) (Additional file 3: Fig S3C). The range of fold changes of early stage LUAD to non-tumor samples was larger than that of late stage vs early stage LUAD samples, which was mainly caused by the higher similarity inside tumor samples. Regrettably, there were few late-stage-specific proteins found in this analysis (Additional file 3: Fig S3C).

### Screening of LUAD prognostic biomarkers

**Fig. 5:**
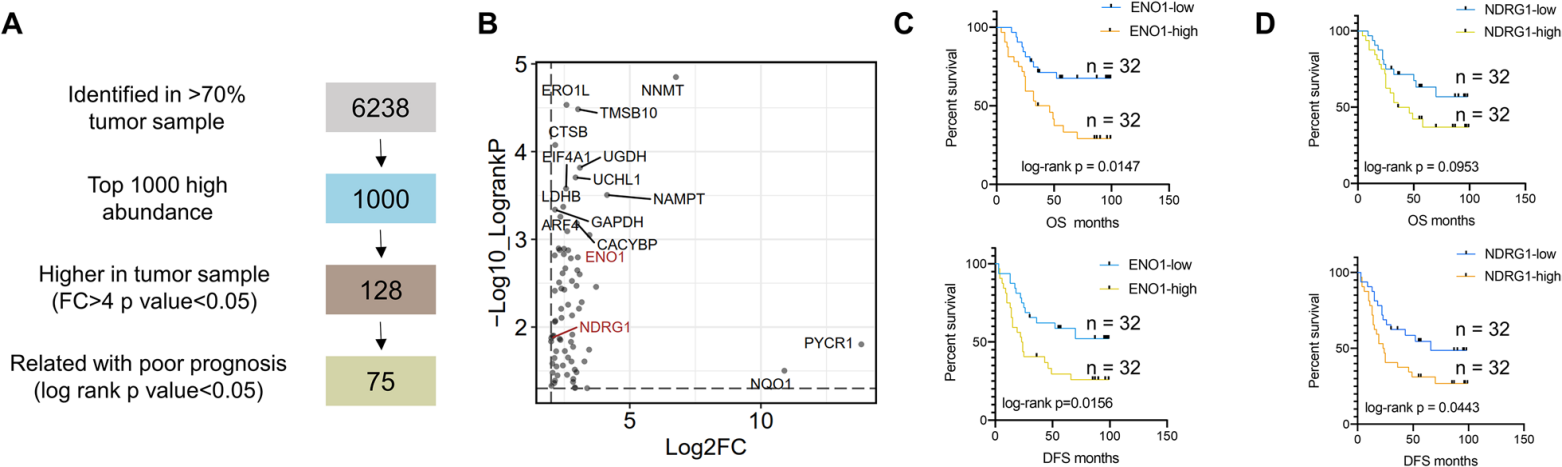
Screening of LUAD prognostic biomarkers. **A** Flow chart of prognostic biomarker selection. **B** Log-rank p values and fold of changes (tumor vs non-tumor) of 75 potential prognostic biomarkers. **C** Kaplan–Meier curve of overall survival (top plot) and disease-free survival (bottom plot) in samples with high expression (red line) and low expression (blue line) of ENO1. **D** Kaplan–Meier curve of overall survival (top plot) and disease-free survival (bottom plot) in samples with high expression (red line) and low expression (blue line) of NDRG1

Non-invasive diagnosis possesses the advantages of convenience and high patients’ compliance. Therefore, we explored new serum prognostic biomarker based on our dataset. Considering that our proteomic data was collected from tissues and there were differences between the protein abundance in tissue and serum, we customized a series of criteria to filter potential poor prognostic biomarkers [19]. Firstly, only proteins identified in more than 70% tumor samples (n = 6,238) and among the top 1,000 high abundant proteins were selected to ensure the detective probability. Secondly, considering the tumor specificity, proteins with fold of change > 4 and *p* < 0.05 were picked out. As a result, among the 128 proteins selected, 75 proteins were correlated with poor prognosis in LUAD (Fig. 5A, B, Additional file 7: Table S2). We chose 2 candidates from the potential biomarkers, ENO1 and NDRG1, for subsequent validation in an independent serum cohort. Alpha-enolase (ENO1) were previously reported to promote metastasis of lung cancer via HGDR and Wnt signaling pathway [34] and higher anti-ENO1 antibody level was associated with better progression-free survival in non-small cell lung carcinoma (NSCLC) patients after surgery [35]. Our ELISA results showed that the high expression of ENO1 was positively correlated with both poor overall survival (OS, *p* = 0.0147) and poor disease-free survival (PFS, *p* = 0.0156) (Fig. 5C). N-myc downstream-regulated gene 1 (NDRG1), encoding a growth and cancer related protein, was confirmed to be overexpressed in lung tumor tissues [36] and correlated with tumor angiogenesis in LUAD patients [37]. In our cohort, the high expression of EDRG1 was significantly correlated with poor DFS (*p* = 0.0443) (Fig. 5D). Meanwhile, the expression level of NDRG1 increased along with the tumor stage (Additional file 4: Fig S4A). Asides from NDRG1, we identified a total of 52 up-regulated proteins and 36 down-regulated proteins altered gradually along with the tumor stage (Additional file 4: Fig S4B), among which IL-1 receptor-associated kinase 1 (IRAK1) and colony stimulating factor 1 receptor (CSF1R) were already reported to be potential drug targets in the therapy of nasopharyngeal carcinoma [38], FGFR1-driven hematological malignancies [39] and sarcoma [40], separately (Additional file 4: Fig S4C). In addition to potential prognostic biomarkers, we also filtered potential early stage diagnostic biomarkers using following criteria (Additional file 5: Fig S5A): 1. The candidate proteins were expressed in more than 70% of the 51 early-stage LUAD samples; 2. The candidate biomarkers had top 1000 high abundances; 3. The candidates were expressed at least fourfold higher in Stage I LUAD than the adjacent tissues (Wilcoxon rank-sum test, multiple hypothesis testing correction, FDR < 0.05); 4. The candidates belong to the HPA secretory database (n = 1,871). As result, a total of 9 potential diagnostic biomarkers (COPA, NAMPT, GPI, CTSB, MIF, SFN, MZB1, PIGR, MUC5B) were filtered out (Additional file 5: Fig S5A, B).

## Discussion

In this study, we conducted a quantitative proteomic analysis of 14 benign lung disease cases and combined their data into our previous 103 paired LUAD proteomic dataset. Our dataset showed that a set of proteins, which mainly belong to cell proliferation and ubiquitin mediated proteolysis pathways, were expressed more frequently in tumor samples. In the comparison of SOL to the non-SOL adenocarcinoma samples, we found that cell proliferation related pathways and NF-kappa B signaling pathway were dominant in SOLs, which promoted the understanding of the clinical poor prognosis in SOL adenocarcinoma patients. Additionally, we uncovered that the hypoxia-induced mitophagy pathway was up-regulated particularly in early stage LUAD, which was the main characteristic of early stage LUAD in our proteome data.

This study provided opportunities for identification of diagnostic and prognostic biomarkers. In the biomarker analysis approach, we chose the proteins identified in over 70% tumor samples, the relative loose criterion mitigated the information loss caused by missing values in the label-free proteomic quantification strategy. In addition, the top 1,000 proteins with high abundance were selected based on the hypothesis that proteins exhibiting higher levels of abundance are more likely to be detected in serum. Apart from the two prognostic biomarkers (ENO1 and NDRG1) we have already validated in independent serum cohort; our analysis also generated a wealth of data resource of potential prognostic and early-stage diagnostic biomarkers of LUAD. Some of the biomarkers listed in this paper were also supported by other researches. For example, the association between the over-expression of ERO1L and the poor survival of LUAD was already verified in patients’ tissue level by immunohistochemical staining [41]. The recent discovery of ERO1L's role in promoting proliferation and metastasis in LUAD [42] also confirmed the reliability of our findings. In addition, among the early-stage diagnostic biomarkers we filtered, PIGR was already reported as lung cancer-related plasma biomarker [43]. MUC5B [44, 45], SFN [46] and MIF [47–49] were detectable in serum and related to lung-associated diseases. MZB [50], CTSB [51, 52], GPI [53], NAMPT [54] were detectable in serum and related to other diseases.

Proteins that gradually up-regulated during LUAD progress and positively correlated with unfavorable prognosis may play essential roles in tumor development, warranting further investigation (Additional file 5: Fig S5B). Within these proteins, we considered IRAK1 and CSF1R as potential pharmacological targets for the treatment of LUAD, based on previous research indicating they could be targeted by FDA-approved drugs, though in other tumor types [38, 40].

Generally speaking, cancer cells were characterized by capability to escape from apoptosis [55]. Higher susceptibility to mitophagy and apoptosis in lung cancer cells has been reported to decrease proliferation [55, 56]. However, we observed a significant upregulation of the mitophagy pathway in early-stage LUAD. Specifically, two up-regulated proteins, Bnip3L and FUNDC1, were outer mitochondrial membrane proteins which could bind directly to LC3 on autophagosome and involved in hypoxia-induced mitophagy [57–59]. The findings revealed the potential role of hypoxia-induced mitophagy in early LUAD, which may act as a transitional biological process in the tumor genesis. Nevertheless, further evidence is required to support this hypothesis.

## Method

### Protein extraction and tryptic digestion

Samples were washed with phosphate buffer saline (PBS) buffer for five times to remove debris and blood. Urea lysis buffer was prepared as following formula: 8 M urea, 100 mM ammonium bicarbonate, added with protease inhibitors and pH 8.0. Samples were cut up and then lysed in lysis buffer for 20 min on ice. Samples were then sonicated for 2 min (sonicated for 3 s and suspended for 3 s) on ice. After centrifuged (21,000 g, 10 min), the supernatants containing soluble proteins were collected. The BCA protein assay was used for the protein concentration measurement. Extracted proteins were reduced in 5 mM dithiothreitol (56 ℃, 30 min) and then alkylated in 15 mM iodoacetamide (room temperature, darkness, 30 min). Alkylation reaction was quenched in 30 mM cysteine (room temperature, 30 min). Proteins were digested with trypsin solution (1:50 w/w, 37 ℃, 16 h) and then desalted by SepPak C18 cartridges. The peptides were dried in vacuum environment of Speed Vac.

### Peptide pre-fractionation by high-pH HPLC

Tryptic peptides were fractioned to 10 fractions by high-pH HPLC before MS/MS detection. Briefly, vacuum-dried peptides were dissolved in buffer A (2% acetonitrile (ACN), pH 9.5) and then loaded on an Xbridge C18 column (4.6 mm × 100 mm, 130A˚,3.5 μm) and eluted at a flow rate of 0.6 mL/min with a 60 min gradient from 0 to 95% buffer B (98% ACN, pH 9.5).

### Nano-LC–MS/MS

Peptide samples were analyzed on Orbitrap Fusion mass spectrometry coupled with an EASY-nLC 1000 LC. Peptides were re-dissolved in mobile phase A (2% ACN and 0.1% formic acid) and then separated in a home-made C18 nano-capillary analytical column with a 60 min gradient from 5 to 80% of buffer B (buffer A: 0.1% formic acid in water; buffer B: 0.1% formic acid in 90% ACN) at a flow rate of 350 nL/min.

The eluted peptides were then analyzed in mass spectrometry at data-dependent acquisition mode. Ions with m/z ranging from 350 to 1300 were acquired for the MS1 full scan by Orbitrap with following parameters: resolution was set to 120,000, the maximal ion injection time (IT) was set to 50 ms and the automatic gain control (AGC) was set to 5 × 10^5^. MS2 acquisition was performed in a top-speed mode with a duty cycle time of 3 s. Precursor ions were fragmented in higher energy collision dissociation (HCD) and then the fragment ions were analyzed in ion trap with following parameters: The maximal IT of MS2 was set as 35 ms. The AGC was set at 7000 and the dynamic exclusion was set as 60 s.

### MS database searching

MS raw files generated by LC–MS/MS were searched using MaxQuant (version 1.6.5.0) software against the same UniProt human proteome database used in the previous study [19]. Research parameters were set as following: digestion type was set as trypsin/P. Max missed cleavages were set as 2. Carbamidomethyl (C) were set as fixed modification. Oxidation (M) and acetylation (Protein N-term) were set as variable modifications.

### Data normalization

Reverse or potential contaminant peptides were removed from MaxQuant search result. Search results of CNHPP cohort and benign lung disease samples were log2 transformed and combined to a matrix including 220 samples. The protein matrix was quantile normalized with R package ‘preprocessCore’. Finally, we used the mean abundance of gene-coding proteins per gene to represent the abundance of the gene and generated a normalized gene level expression matrix. Except for analysis mentioned particularly, we performed differential protein analysis using the proteome matrix with less than 50% missing values and without missing value imputation. Considering that many tumor related proteins didn’t detected in most non-tumor samples, to facilitate biomarker filtering, for early-stage/late-stage special proteins analysis and prognostic/diagnostic biomarker filtering analysis, we used proteome matrix with less than 70% missing values in whole tumor (n = 103) or early-stage tumor samples (n = 51) and imputed the missing values with minimum value of original matrix (n = 220, containing 11,234 genes).

### Protein subcellular location summary

The protein subcellular location database was downloaded from The Human Protein Atlas website. Some subcellular groups were merged based on the information of cellular & organelle proteomic module on the website [60] (https://www.proteinatlas.org/humanproteome/subcellular).

### Pathway enrichment analysis

Over Expression Analysis was conducted for LRPs, NDRPs, Stage I unique up-regulated proteins, and shared up-regulated proteins through WebgestaltR [61]. Gene Set Enrichment Analysis (GSEA) [62] was conducted for the comparison between SOL and non-SOL adenocarcinoma samples.

### Protein network analysis

LRPs were introduced to and analyzed in STRING website [63]. The STRING output was visualized in Cytoscape (v3.9.1). Protein cluster analysis were conducted by the plug-in MCODE of Cytoscape [64, 65].

### Differentially expressed protein analysis

Wilcoxon sum rank test was applied for differentially expressed protein analysis between every two groups. Fold of change per gene was defined as the ratio of median expression value of the gene in each group.

### CERES score calculation

CERES data (CRISPR_gene_effects.csv, 2021.06) was downloaded from DepMap Portal [23]. For each gene, the median of CERES scores of the gene in 88 lung adenocarcinoma cell lines was calculated.

### Supplementary Information

**Additional file 1: Figure S1.** Sample information and data quality control. A Detail disease information for benign lung disease samples. B Correlation between CNHPP dataset and re-normalized proteome dataset for the 103 paired LUAD and NATs. C Sample distribution before (left) and after (right) quantile normalization of all the 220 samples. Benign disease samples were marked with red. D Number of quantified proteins of per benign lung disease, NAT and LUAD samples. E Number of total quantified proteins of all the benign lung disease, NAs and LUAD samples in subcellular level.

**Additional file 2: Figure S2.** A Heatmap of up-regulated expressed proteins (fold of change > 2, p value < 0.05) in benign lung disease and NAT groups. B ORA pathway enrichment results for protein sub-clusters of LRPs.

**Additional file 3: Figure S3.** A Distribution of the expression of three ‘Stage I unique up-regulated proteins’ belonging to mitophagy pathway in non-tumor, early stage and middle and late stage tumor groups (*p < 0.05. **p < 0.01. ***p < 0.001). B Kaplan–Meier curve of overall survival in samples with high expression (red line) and low expression (blue line) of FUNDC1. C Comparison of fold-changes in early stage (Stage I vs non-tumor) or late stage (Stage II/III/IV vs Stage I) LUAD samples. Red dots: proteins up-regulated (fold of change > 2, p < 0.05) in both early stage or late stage LUAD groups. Blue dots: proteins down-regulated (fold of change < 1/2, p < 0.05) in both early stage or late stage LUAD groups. Orange dots: proteins up-regulated (fold of change > 2, p < 0.05) in only early stage group. Pink dots: proteins up-regulated (fold of change > 2, p < 0.05) in only late-stage LUAD group. Grey dots: proteins without significant change (p > 0.05) in both early stage or late-stage LUAD groups.

**Additional file 4: Figure S4.** A Top panel: NDRG1 expression level in non-tumor, early stage and middle and late stage tumor groups. Bottom panel: Kaplan–Meier curve of overall survival in samples with high expression (red line) and low expression (blue line) of NDRG1. B Top panel: 52 proteins up-regulated gradually along with the increasing of tumor stages. Bottom panel: 36 proteins down-regulated gradually along with the increasing of tumor stages. C Top panel: IRAK1 expression level in non-tumor, early stage and middle and late stage tumor groups. Bottom panel: Kaplan–Meier curve of overall survival in samples with high expression (red line) and low expression (blue line) of IRAK1. D Top panel: CSF1R expression level in non-tumor, early stage and middle and late stage tumor groups. Bottom panel: Kaplan–Meier curve of overall survival in samples with high expression (red line) and low expression (blue line) of CSF1R. (*p < 0.05. **p < 0.01. ***p < 0.001).

**Additional file 5: Figure S5.** A Flow chart of potential serum diagnostic biomarker selection. B FDR of Wilcoxon rank rum test and fold of changes (early-stage tumor vs non-tumor) of 9 potential early-stage prognostic biomarkers.

**Additional file 6: Table 1.** Quantile normalized protein expression for all individual samples.

**Additional file 7: Table 2.** List of potential LUAD prognostic biomarkers.

## Data Availability

All protein identification and normalized protein quantification data for all individual samples can be found in Additional file 6: Table S1. The MS/MS raw data is available from the corresponding author on request.
