# Supplementary figures and images for: Prognostic biomarker discovery based on proteome landscape of Chinese lung adenocarcinoma
Source: Clin Proteomics. 2024 Jan 5;21:2. doi: 10.1186/s12014-023-09449-2 (PMC10768252; doi:10.1186/s12014-023-09449-2)

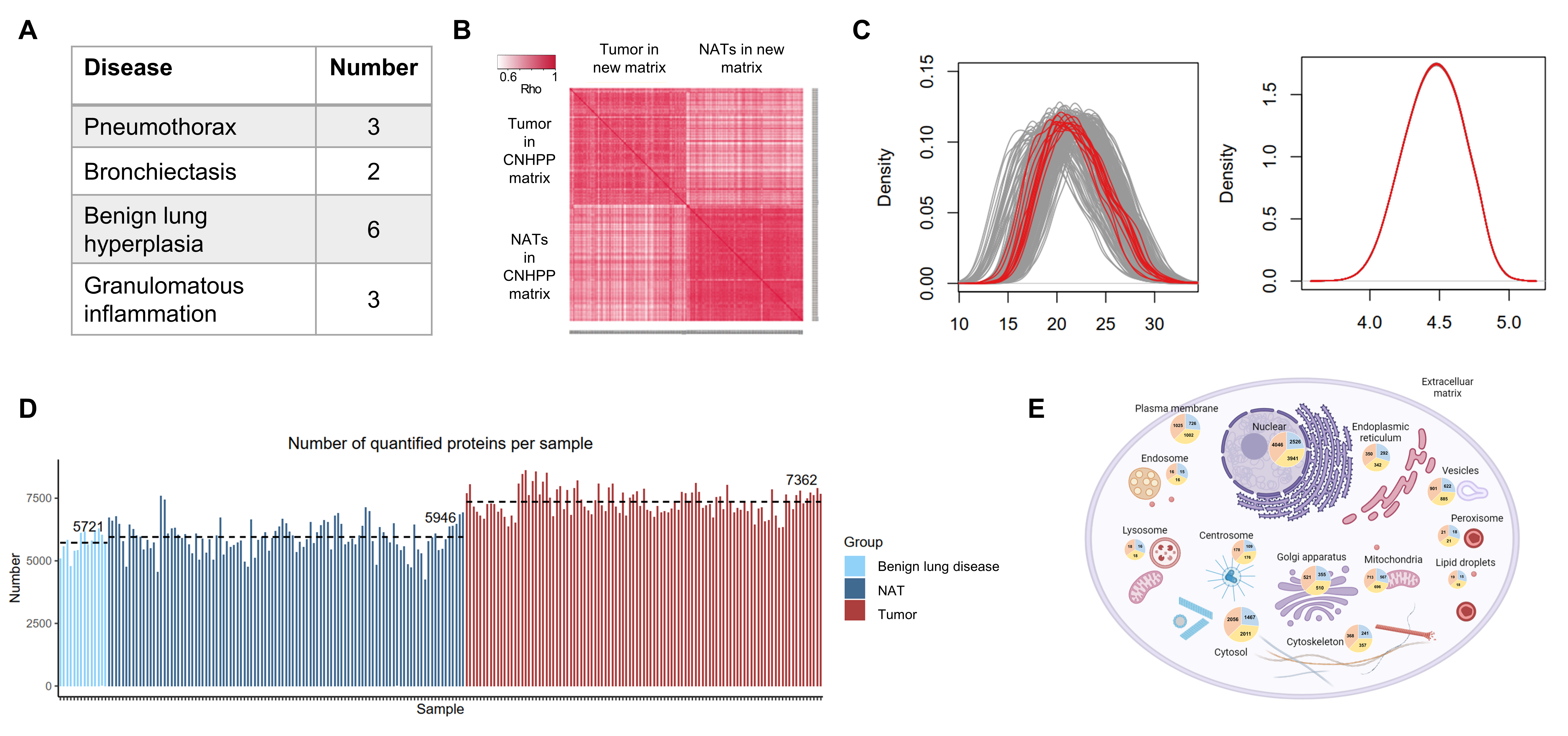

Supplement: Supplementary file 1 — Additional file 1: Figure S1. Sample information and data quality control. A Detail disease information for benign lung disease samples. B Correlation between CNHPP dataset and re-normalized proteome dataset for the 103 paired LUAD and NATs. C Sample distribution before (left) and after (right) quantile normalization of all the 220 samples. Benign disease samples were marked with red. D Number of quantified proteins of per benign lung disease, NAT and LUAD samples. E Number of total quantified proteins of all the benign lung disease, NAs and LUAD samples in subcellular level. [file 12014_2023_9449_MOESM1_ESM.tif]

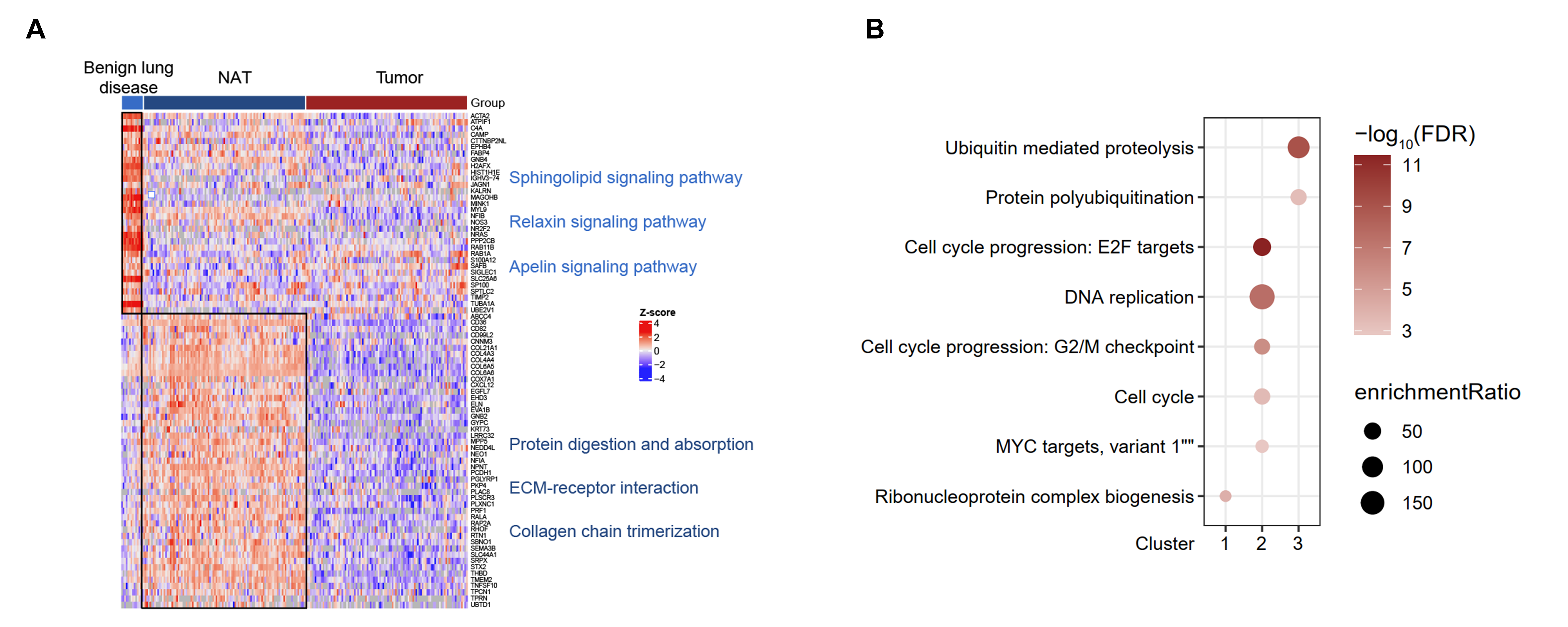

Supplement: Supplementary file 2 — Additional file 2: Figure S2. A Heatmap of up-regulated expressed proteins (fold of change > 2, p value < 0.05) in benign lung disease and NAT groups. B ORA pathway enrichment results for protein sub-clusters of LRPs. [file 12014_2023_9449_MOESM2_ESM.tif]

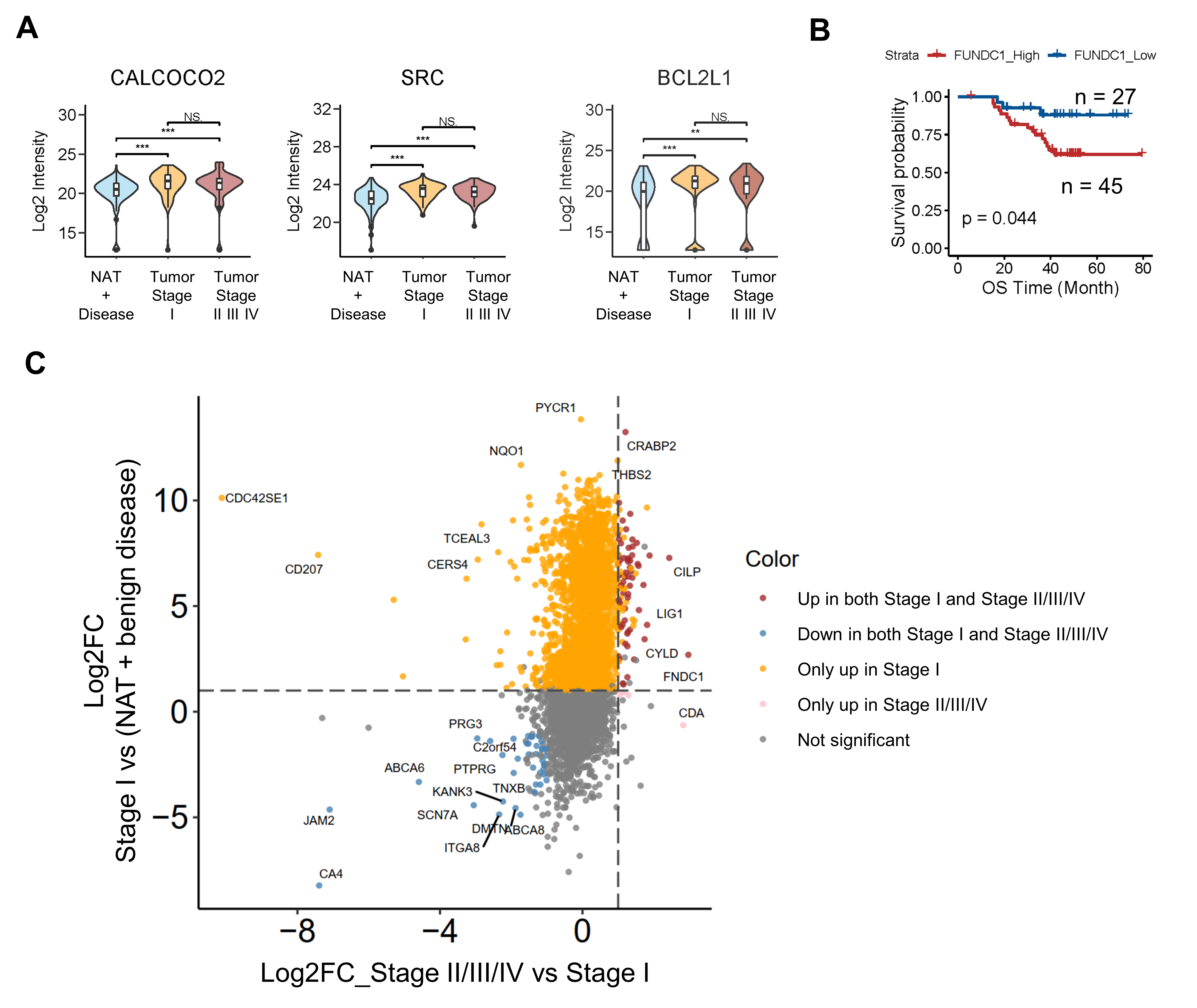

Supplement: Supplementary file 3 — Additional file 3: Figure S3. A Distribution of the expression of three ‘Stage I unique up-regulated proteins’ belonging to mitophagy pathway in non-tumor, early stage and middle and late stage tumor groups (*p < 0.05. **p < 0.01. ***p < 0.001). B Kaplan–Meier curve of overall survival in samples with high expression (red line) and low expression (blue line) of FUNDC1. C Comparison of fold-changes in early stage (Stage I vs non-tumor) or late stage (Stage II/III/IV vs Stage I) LUAD samples. Red dots: proteins up-regulated (fold of change > 2, p < 0.05) in both early stage or late stage LUAD groups. Blue dots: proteins down-regulated (fold of change < 1/2, p < 0.05) in both early stage or late stage LUAD groups. Orange dots: proteins up-regulated (fold of change > 2, p < 0.05) in only early stage group. Pink dots: proteins up-regulated (fold of change > 2, p < 0.05) in only late-stage LUAD group. Grey dots: proteins without significant change (p > 0.05) in both early stage or late-stage LUAD groups. [file 12014_2023_9449_MOESM3_ESM.tif]

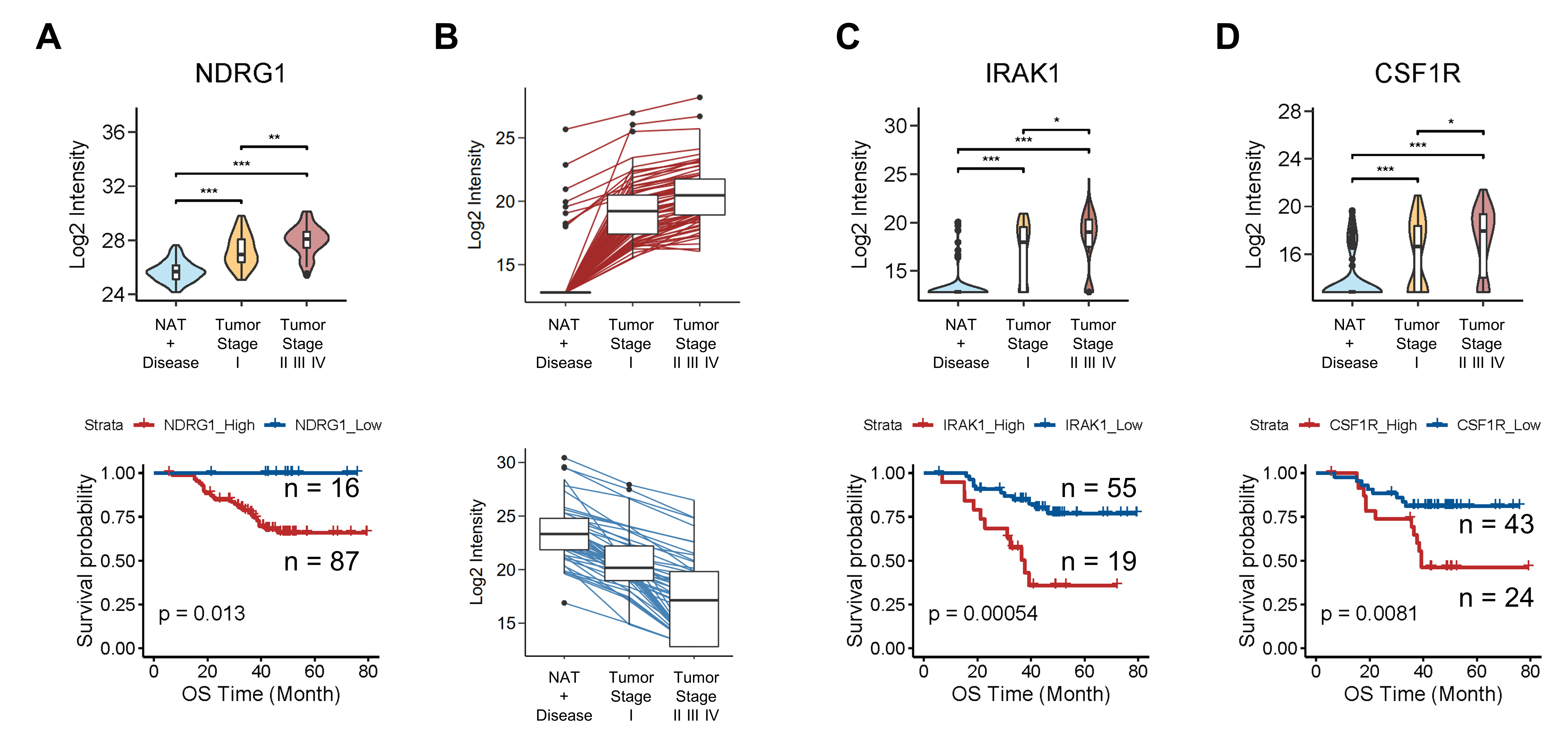

Supplement: Supplementary file 4 — Additional file 4: Figure S4. A Top panel: NDRG1 expression level in non-tumor, early stage and middle and late stage tumor groups. Bottom panel: Kaplan–Meier curve of overall survival in samples with high expression (red line) and low expression (blue line) of NDRG1. B Top panel: 52 proteins up-regulated gradually along with the increasing of tumor stages. Bottom panel: 36 proteins down-regulated gradually along with the increasing of tumor stages. C Top panel: IRAK1 expression level in non-tumor, early stage and middle and late stage tumor groups. Bottom panel: Kaplan–Meier curve of overall survival in samples with high expression (red line) and low expression (blue line) of IRAK1. D Top panel: CSF1R expression level in non-tumor, early stage and middle and late stage tumor groups. Bottom panel: Kaplan–Meier curve of overall survival in samples with high expression (red line) and low expression (blue line) of CSF1R. (*p < 0.05. **p < 0.01. ***p < 0.001). [file 12014_2023_9449_MOESM4_ESM.tif]

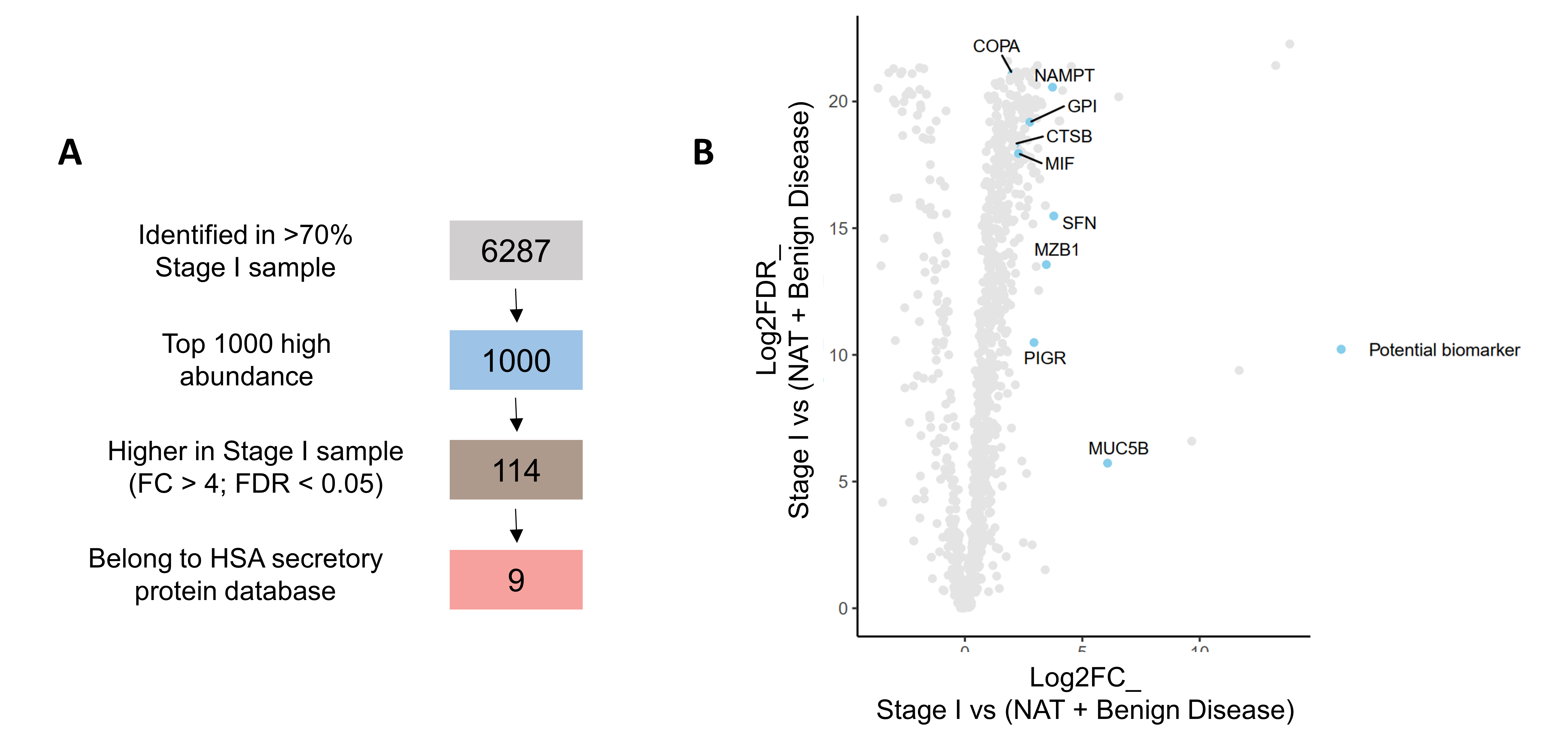

Supplement: Supplementary file 5 — Additional file 5: Figure S5. A Flow chart of potential serum diagnostic biomarker selection. B FDR of Wilcoxon rank rum test and fold of changes (early-stage tumor vs non-tumor) of 9 potential early-stage prognostic biomarkers. [file 12014_2023_9449_MOESM5_ESM.tif]
